# Engaging with HIV care systems: why space, time and social relations matter

**DOI:** 10.1136/sextrans-2017-053173

**Published:** 2017-07-23

**Authors:** Karina Kielmann, Fabian Cataldo

**Affiliations:** 1 Institute for Global Health & Development, Queen Margaret University, Edinburgh, UK; 2 Dignitas International, Zomba, Malawi

**Keywords:** HIV, AFRICA, TREATMENT, QUALITATIVE RESEARCH

Care trajectories of people living with HIV (PLHIV) in Southern and Eastern Africa have drastically changed over the past two decades as a result of significant funding to support health systems’ responses to HIV. Global expansion of access to diagnostic procedures and treatment has extended and improved the health and well-being of PLHIV, and modified the scope of HIV care. In the absence of treatment, testing for HIV once represented a critical, yet stand-alone, moment in an uncertain and fragmented care pathway. Early emphasis on voluntary testing acted as a kind of ‘**confessional technology**’:^1^ a means to ‘know your status’ and to contribute to the management of collective risk. As antiretroviral therapy (ART) became more widely available, HIV testing came to be seen as the gateway for timely access to treatment, with the weight of responsibility for ensuring the care continuum falling to health providers. Global strategies to meet specific HIV-related targets, often described in the bureaucratic language of service delivery—roll-out, scale-up, decentralisation and integration—entailed reconfigurations of the health workforce implementing HIV programmes. At the same time, securing commitment to lifelong adherence to ART from PLHIV translated the promise of universal test-and-treat programmes in many high-burden countries into renewed emphasis on responsibilisation of patients and their families.^2^ The special issue brings together a series of papers that provide critical and timely inquiry into a specific moment in the historical trajectory of HIV care. As such, it is worthwhile recalling how spatial, temporal and relational parameters of the current drive towards universal test-and-treat models have evolved. In the course of the past two decades, HIV testing and counselling procedures have undergone substantial revision. Tests are rapid and routinised, and take place outside of the clinic: new sites for testing represent a continuum of social spaces, spanning the intimacy of homes and domestic arrangements as well as the relative anonymity of mobile testing facilities and mass public testing campaigns. Rapid testing technologies, while reducing delays between the decision to test, actual testing and the disclosure of test results, may paradoxically deprive individuals of the time required to digest a test result and its consequences, and to think about when and with whom to share test results. The onus of diagnosis and disclosure has shifted from voluntary to provider-initiated and in some contexts, self-testing. While rapid self-testing is hailed as a means for individuals to gain ownership and confidence in the technology, the prerogative to test in many sub-Saharan African contexts remains entangled with the imperatives of large screening programmes. Although systems delays in the HIV cascade of care have been reduced, these may be accompanied by unwelcome short cuts in humane care, as witnessed, for example, in the transition from individualised counselling interactions to prescriptive instructions or group health education lectures. When guidelines are rigidly applied without due consideration for individual circumstances, the standardisation of tasks can have detrimental effects on patient care.^3^

HIV testing has become embedded within the logic of the care continuum,^4^ which sees individuals progressing sequentially from testing and diagnosis to being linked to HIV care, initiating ART, remaining engaged in care and ultimately achieving viral suppression. From a social and ‘lived experience’ perspective, this logical progression is not self-evident. Health-seeking is never a one-off event but part of a longer trajectory of engagement or disengagement with the health system over a lifetime. Being a 'good patient' may imply knowing how to negotiate spaces and the temporal logic of clinical care pathways, yet these behaviours are acquired rather than assumed. Current research on 'implementation barriers' and challenges with loss to follow up reveal the subtlety of factors influencing individual patients’ health literacy, agency in decision-making and capacity to assume the 'good patient' role.^5^

These ongoing changes to the way HIV care is provided and organised have forced health systems to reconfigure the identities, roles and responsibilities of all actors involved. Health providers are under pressure to meet new targets^6^ for initiating and retaining individuals on treatment and may resort to tactics that many researchers have described as bordering on coercive.^7 8^ Decentralised care, including task-shifting and task-sharing strategies have been employed to respond to the lack of human resources for health and to increase the influx of patients, yet critics argue that there are ‘**no panacea**’ for the ‘**systemic shortcomings of weak health systems**’.^9^ Surprisingly little attention is given to the intrinsically hierarchical nature of social divisions in diverse, real life health systems, the impact of these on staff working relations and patient-provider interactions, and consequently, the relative success of decentralised human resources for HIV care strategies, including task-shifting and creation of new cadres of care workers to support formal health staff.^10^

The emphasis on community health workers and lay engagement in HIV care appears promising in settings where there are strong traditions of community-based social movements and mobilisation around healthcare.^2^ However, in many settings where there are limited spaces for individuals to actively engage with, and shape health services, linkages are ill-defined, job descriptions for lay health workers are loose, and task shifting activities are poorly integrated into the health system or diverted from their intended purpose.^11^ ‘Expert patients’, for example, are increasingly enlisted in many HIV programmes to support testing and long-term engagement in care. By caring for HIV clients and for themselves through learnt tasks that are part of the clinical regimen and the wider clinical working environment, expert patients in high-burden settings often tend to define themselves through vertical (professional hierarchy) rather than horizontal (solidarity and empathy with other patients) links.^12^ Perhaps unsurprisingly in contexts of economic scarcity and a widespread HIV epidemic, expert patients’ goals of professional mobility within the health system becomes more important than therapeutic solidarity with their peers and clients.

Despite the onus of treatment initiation and adherence monitoring being placed on health workers, a parallel move to hand over responsibility for testing and interpreting test results to patients is in evidence, resonating more broadly with the move towards patient self-management. Treatment ‘literacy’ approaches espoused by large-scale programmes to deliver HIV care since the mid-2000s^13^ have hailed the importance of patient-centred and rights-based discourses around patient empowerment and participation, and the dawn of a ‘**new kind of relationship or contract between providers and patients.'** 14 Patient-centred approaches are promoted as an efficient way to ensure the care continuum across the wide range of actors who respond to the needs of PLHIV across the life span of chronic illness, yet links across the informal/formal, public/private and clinic/community divides tend to be tenuous, fragmented or non-existent in actual practice.^15^

A number of well intended patient-centred interventions contain deeply rooted assumptions about individual agency in a rapidly changing political context and apparatus of HIV care. Some interventions instrumentalise social relations of care in ways that are fundamentally at odds with the larger structural forces framing working relationships within the health system, and within communities affected by HIV more broadly. These relationships may be compromised through an awareness of acute imbalances and inequities in resources, and social and symbolic capital. Test-and-treat approaches in the prevention of mother-to-child transmission of HIV (PMTCT), for example, place responsibility on women to initiate treatment, provide postnatal care for their infant at precise time intervals, disclose their HIV status to their families and engage their sexual partners in the process of care. Beyond testing an individual pregnant woman, these practices increasingly test social and moral expectations in women’s relationships with men and invoke their moral duty as mothers in relation to the unborn child.^16^ Couple counselling and testing approaches, similarly, are often premised on an idealised model of conjugal relations and couple communication, one that may hold up only weakly against the realities of men's and women’s lives fragmented through economic hardship, illness, migration and death.^17 18^

The advent and universal expansion of new diagnostic and treatment modalities is reshaping care practices in settings which traditionally had little access to basic medical necessities, let alone prognostic technologies and expensive treatment regimens. This quickly moving landscape of HIV care necessitates closer examination, specifically of the bridges and disjunctures between the local dynamics of provider-patient and community interactions and the wider goals of these programmes at each critical step of the HIV care pathway. Global health imperatives such as the roll-out of ART and new strategies to accelerate testing and treatment initiation have been translated into ambitious implementation plans that often rely on existing healthcare providers and fragile health systems. As front-line care providers become the interface between large-scale policy aspirations and local programme implementation, they also become invested with a liability to comply with model targets and operate within more complex, technical systems of care. In line with the developments we describe above, the papers in this special issue offer nuanced and engaging examples of the ways in which social and moral relations are at play, and, to some extent, act to temper ambitions of universality within evolving configurations of HIV care.
